# Therapeutic targets in membranous nephropathy: plasma cells and complement

**DOI:** 10.1093/ckj/sfae243

**Published:** 2024-08-13

**Authors:** Nicola M Tomas

**Affiliations:** III. Department of Medicine, University Medical Center Hamburg-Eppendorf, Hamburg, Germany; Hamburg Center for Kidney Health, University Medical Center Hamburg-Eppendorf, Hamburg, Germany; Hamburg Center for Translational Immunology, University Medical Center Hamburg-Eppendorf, Hamburg, Germany

**Keywords:** autoantibodies, complement, glomerulonephritis, membranous nephropathy, podocytes

## Abstract

Membranous nephropathy (MN) is an antibody-mediated autoimmune disease and the most common cause of nephrotic syndrome in adults. The discovery of phospholipase A2 receptor 1 (PLA2R1) as the first target antigen in patients with MN 15 years ago has led to a paradigm shift in the pathobiological understanding of this disease. Autoantibodies against PLA2R1 as well as thrombospondin type-1 domain-containing 7A, the second identified antigen in adults, were shown to be disease-causing and act through local activation of the complement system, primarily via the classical and lectin pathways. These findings indicate that both plasma cells, the main source of antibodies and autoantibodies, as well as the complement system, the main pathogenic effector mechanism in MN, are rational and pathogenesis-based treatment targets in MN. This review summarizes pathomechanistic and clinical evidence for and against plasma cell– and complement-targeted treatments in MN.

## INTRODUCTION

Membranous nephropathy (MN) is an autoimmune kidney disease and a common cause of nephrotic syndrome in adults. The histological hallmarks are a thickening of the glomerular basement membrane in light microscopy and granular deposition of immunoglobulin G (IgG) and complement in immunofluorescence microscopy, suggesting a pathogenic role of both autoantibodies and the complement system in this disorder. Lately, tremendous progress with regard to the pathomechanistic understanding of the disease has been achieved, initiated by the discovery of phospholipase A2 receptor 1 (PLA2R1) as a target antigen in ≈70% of patients with MN in 2009 [1]. In 2014, thrombospondin type-1 domain-containing 7A (THSD7A) was identified as the second autoantigen in MN [2]. Since then, numerous antigens have been identified (reviewed in Hoxha *et al.* [3] and Caza *et al.* [4]), most recently leading to a new, antigen-based classification of the disease [5]. Importantly, anti-PLA2R1 and anti-THSD7A autoantibodies are not simply biomarkers with outstanding performance with regard to diagnosis, monitoring and prediction of remission and relapse [6–11], but in fact cause MN through interaction with the respective target antigen at the podocyte membrane [12, 13]. Disappearance of anti-PLA2R1 autoantibodies from the blood usually precedes clinical remission by several weeks to months [14], suggesting that achieving immunological remission should be the primary treatment goal in MN patients, at least in the context of anti-PLA2R1 antibody positivity.

**Figure 1:**
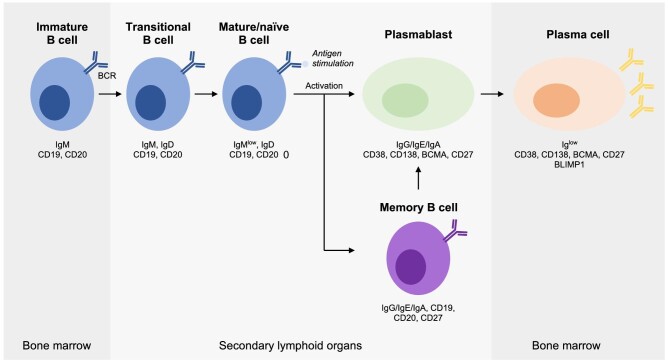
Overview on B cell biology. Members of the B cell family involve, among others, immature, transitional, mature, naïve and memory B cells as well more differentiated cells such as plasmablasts and plasma cells. These cells show distinct expression patterns of certain surface molecules, enabling identification of these cells, but also therapeutic targeting.

**Figure 2:**
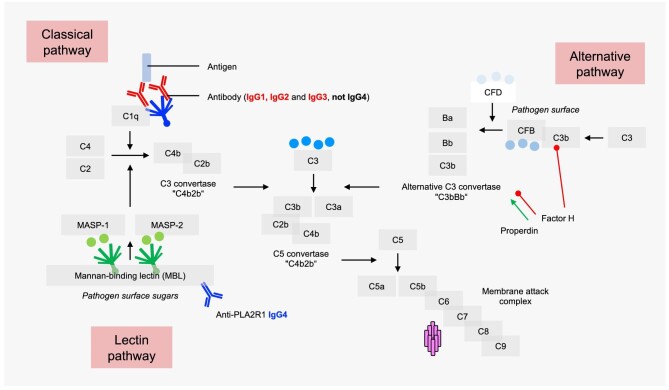
Overview of the complement system. The complement system can be initiated by three different activation pathways: classical, lectin and alternative. All lead to the formation of a C3 convertase, anaphylatoxins like C3a and C5a and the formation of the membrane attack complex.

Several experimental studies have recently shed light on the role of autoantibodies and the complement system in MN. The pathogenicity of the involved autoantibodies, triggering activation of the complement system at the podocyte membrane, suggests both plasma cells (the main source of antibodies and autoantibodies; Fig. 1) and the complement system (an antibody effector mechanism leading to target cell injury; Fig. 2) as potential therapeutic targets for patients with MN. However, until today there have been no clinical trials published that investigated the treatment efficacy of these two strategies. This article summarizes recent experimental and clinical evidence in favour of and arguing against plasma cell- and complement-targeted therapies in MN.

## PATHOGENESIS OF MN: ROLE OF B AND PLASMA CELLS

B cells, a crucial component of the adaptive immune system, play a vital role in the generation of plasma cells and production of antibodies. B cells originate from haematopoietic stem cells in the bone marrow and undergo a rigorous selection process to avoid recognition of self-antigens, thereby preventing autoimmunity. Once mature, naïve B cells circulate in the peripheral blood and secondary lymphoid organs, where they encounter antigens. Upon recognition of their cognate antigen through their B cell receptors (BCRs), which are membrane-bound immunoglobulins corresponding to the antibody that is being generated and secreted by this particular B cell clone, B cells undergo activation, which requires additional signals from helper T cells and specific cytokines. Upon activation, B cells proliferate and differentiate into two main types of cells: plasma cells and memory B cells (Fig. 1). Plasma cells are the effector cells that produce and secrete large quantities of antibodies, also known as immunoglobulins, while memory B cells are essential for long-term immunity, allowing for a rapid and robust response upon subsequent re-exposures to the same antigen. Plasmablasts, which can also produce large amounts of antibodies within a short period of time, are cells at an intermediate stage between activated B cells and fully differentiated plasma cells. The differentiation into plasma cells typically occurs in the germinal centres of lymph nodes, where B cells undergo class switching and somatic hypermutation, leading to the generation of high-affinity IgG antibodies. The lifespan of plasma cells can vary; some are short-lived, providing immediate protection, while others migrate to the bone marrow, where they can survive for years, continually secreting antibodies and providing long-term immunity (Fig. 1). All these B cell subtypes express a distinct pattern of surface molecules that can be used to identify and classify these cells. For example, CD20 is mainly expressed on immature, naïve, mature and memory B cells, and is typically lost on plasmablasts and plasma cells, while the B cell maturation antigen (BCMA) has its highest expression on plasmablasts and plasma cells. CD19, in contrast, is expressed on B cells of all maturation stages with the exception of plasma cells, where CD19 is typically considered to be absent or very low. Similarly, BCR has its highest expression on B cell subtypes like naïve, activated and memory B cells, but is less expressed on plasmablasts and hardly expressed on plasma cells at initial phases of differentiation.

Normally, autoreactivity of antibodies is prevented, but non-autoreactive precursor cells can develop into autoreactive B cells by somatic mutation of a BCR during cell proliferation in germinal centres [15]. The precise mechanisms of loss of tolerance and development of autoimmunity have not been resolved. In MN, factors like genetic predisposition [16], antigen expression by tumours [17], exposure to fine particulate matter or toxins [18, 19], conjunction of microbials with the autoantigen [20] and, possibly, molecular mimicry may lead to a break in tolerance and autoantibody generation with subsequent binding of these autoantibodies to the podocyte membrane [21]. Together, their essential role in autoantibody generation suggests plasma cells as a rational therapeutic target in antibody-mediated diseases like MN.

Several trials have been completed that investigated B cell–targeted treatments in MN. The first one was the GEMRITUX trial (NCT01508468), which compared supportive therapy alone with supportive therapy in combination with rituximab (2 × 375 mg/m^2^) [22]. The primary endpoint was the rate of complete or partial remission at 6 months of follow-up. No difference was seen between the two groups after 6 months. However, at the end of the observational extension period, 65% and 34% were in complete or partial remission in the rituximab and supportive care only groups, respectively, suggesting that the primary endpoint at 6 months was chosen too early to detect the difference between the two groups. The MENTOR trial (NCT01180036) compared cyclosporine with rituximab (2 × 1 g at study start, potentially repeated at 6 months depending on the response) and found complete or partial remission rates of 20% and 60%, respectively, after 2 years of follow-up [8]. This trial confirmed the high rates of relapse in patients after discontinuation of treatment with calcineurin inhibitors. The STARMEN trial (NCT01955187) compared a cyclic alternating treatment with steroids and oral cyclophosphamide with a sequential treatment of tacrolimus and, 6 months later, a single dose of rituximab [23]. The primary endpoint of complete or partial remission at 24 months was achieved in 84% and 58% using the cyclic regimen and the tacrolimus–rituximab scheme, respectively. The weak performance of rituximab in this study is likely explained by the combination of a delayed treatment start of rituximab (thereby delaying the reduction in pathogenic autoantibodies) and a low dose of rituximab (1 g in total). The Ri-CYCLO trial (NCT03018535) compared the cyclic regimen with steroids and cyclophosphamide with 2 × 1 g of rituximab [24]. The primary endpoint was complete remission at 12 months, which was achieved more often in patients receiving the cyclic regimen (32% versus 16%). However, the secondary endpoint of complete or partial remission at 24 months was achieved in 81% and 85% in the two groups. Collectively, these studies pointed to rituximab as a first-line treatment option in the Kidney Disease: Improving Global Outcomes guidelines for glomerular diseases for many patients in 2021 [25].

Obinutuzumab is a second-generation monoclonal anti-CD20 antibody that has been optimized to eliminate B cells by means of glycoengineering of antibody Fc [26]. It has anecdotally been used in patients with MN both as initial therapy as well as secondary therapy after failure of other immunosuppressive treatments [27, 28]. Several studies are currently registered investigating obinutuzumab in patients with MN: the ORION trial (NCT05050214) is a phase 2 trial looking at the efficacy and safety of obinutuzumab in patients that are either rituximab resistant or rituximab dependent. The REMIT trial (NCT06120673) and the MAJESTY trial (NCT04629248) are phase 3 studies investigating the efficacy of obinutuzumab as an initial treatment in patients with newly diagnosed MN. These studies will shed light on whether optimized B cell depletion can achieve higher remission rates than rituximab.

**Figure 3:**
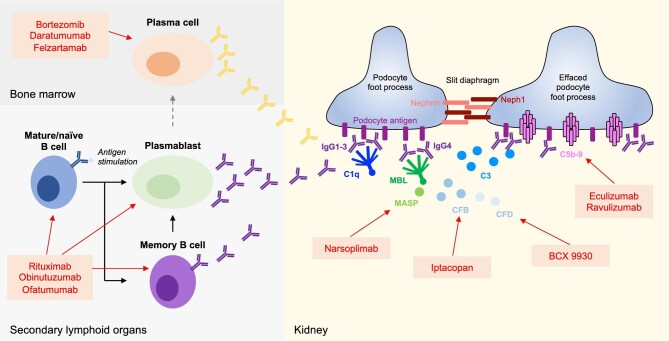
Pathogenesis-based treatment options in membranous nephropathy. Autoantibodies are generated by B cells, memory B cells, plasmablasts and plasma cells in secondary lymphoid organs and the bone marrow. These autoantibodies reach the kidney via the circulation, bind to the target podocyte antigen and induce complement activation mainly via the classical and lectin pathways. This (and potentially other complement-independent mechanisms) leads to podocyte foot process effacement and proteinuria. Several B cell–, plasma cell– and complement-targeted molecules (red) are under investigation or have been investigated in patients with MN.

Autoantibody levels typically decrease after rituximab therapy, although CD20 is not expressed on plasma cells, indicating that short-lived plasmablasts are an important source of antibodies in MN and that elimination of their CD20^+^ precursor B cells indirectly depletes CD20^−^ plasmablasts, leading to disease amelioration (Fig. 3, left). However, 15–40% of patients do not show partial or complete remission in response to rituximab, suggesting that autoantibodies in these cases may be majorly generated by CD20^−^ cells such as plasma cells. Thus the primary targeting of plasma cells represents a promising therapeutic strategy for many patients with MN. In this direction, bortezomib, a proteasome inhibitor, and daratumumab, a monoclonal antibody against CD38, are plasma cell–depleting agents that have anecdotally been used to treat refractory cases of MN with some success [29–32].

## PATHOGENESIS OF MN: ROLE OF COMPLEMENT

The complement system can be initiated via three different pathways: classical, lectin and alternative (Fig. 2). The classical pathway is activated by binding of C1q to the Fc part of antibody complexes, leading to cleavage of C2 and C4, which enables assembly of the classical/lectin C3 convertase C4bC2b. The lectin pathway is initiated by pattern recognition molecules such as mannan-binding lectin (MBL), which bind to MBL-associated serine proteases (MASPs). This also induces cleavage of C2 and C4 and consequently the formation of C4bC2b. The alternative pathway is constantly activated by slow hydrolysis of C3, which, in the presence of complement factors B (CFB) and D (CFD), leads to formation of the alternative C3 convertase C3bBb. Both the classical/lectin and alternative C3 convertases cleave fluid phase C3, and subsequent progression results in assembly of the classical and alternative C5 convertases, resulting in cleavage of C5 and formation of the membrane attack complex C5b-9. C5b-9 builds a pore in the cell membrane, leading to (sub)lytic damage to the target cell. While the detection of complement components such as C3 and C5b-9 in biopsies from patients with almost all forms of MN is beyond doubt, the activation pathways of the complement system in the context of MN have long been a matter of debate.

As MN is an antibody-mediated disease, it appears reasonable to assume that activation of the complement system occurs by the classical pathway. However, autoantibodies against PLA2R1, and also THSD7A, are predominantly of the IgG4 subclass, the IgG subclass with the least C1q binding capacity and thus limited ability to activate the classical pathway [33]. In line with this, C1q was historically described to be absent in glomeruli of MN patients [34]. Other studies found variable frequencies of C1q deposition [35–38] and the presence of C1q-binding IgG1, 2 or 3 in both serum and glomeruli from MN patients [39–42]. The central complement component C3 and the membrane attack complex C5b-9 are usually identified in glomeruli of MN patients, indicating complement activation without allowing conclusions on the initiating pathway(s) [43, 44]. MBL, an initiator of the lectin pathway, is also detected in the glomeruli of patients with MN [45–47]. In a more recent study, anti-PLA2R1 IgG4 with distinct glycosylation patterns was shown to directly bind MBL *in vitro*, thereby activating the complement system via the lectin pathway (Fig. 3, right) [48]. Thus this study provided a new mechanism of complement activation by IgG4, explaining the absence of C1q but the presence of C4d in several studies [47, 49]. Stainings for properdin, CFB and complement factor H (CFH), key components of the alternative pathway, are also regularly positive in MN kidney biopsies [38, 50]. Whether the alternative pathway is activated independently or running via an amplification loop is currently unknown.

Another recent study systematically dissected the activity of the three different complement activation pathways by combining a proximity ligation assay to visualize the classical/lectin and alternative complement convertases in glomeruli with immunofluorescence techniques. The classical/lectin convertase was positive in all investigated patients with PLA2R1- and THSD7A-associated MN, and the alternative convertase was positive in two-thirds of the patients [51]. Upstream of the classical/lectin convertase, C1q was the predominant complement component present in the tissue and all investigated patients had glomerular deposits of C1q-binding IgG1, 2 and 3, in accordance with a major role of classical pathway activation in MN (Fig. 3, right). In line with the previous study demonstrating lectin pathway activation by anti-PLA2R1 IgG4 [48], the degree of IgG4 deposition strongly correlated with activation of the lectin pathway. Moreover, in the first antigen-specific autoimmune model of MN, complement-deficient mice as well as mice treated with a C3-silencing small interfering RNA had less proteinuria, less podocyte foot process effacement and more preserved slight diaphragm proteins [51]. Later, an essential role of the complement system could also be shown in a model of PLA2R1-specific MN [52]. Collectively, these studies indicate that in MN, the complement system is activated via multiple pathways and that it plays a pathogenic role in the development of proteinuria and podocyte damage.

## PLASMA CELL– AND COMPLEMENT-TARGETED TRIALS IN MN

Several molecules aimed at plasma cell depletion and inhibition of the complement system are or have been investigated in clinical trials (Table 1). The only published trial in this regard is the M-PLACE trial (NCT04145440), a phase 1b/2a study investigating felzartamab, a monoclonal antibody against CD38 on plasma cells, in 31 patients with PLA2R1-associated MN (Fig. 3, left). It included patients with newly diagnosed or relapsed PLA2R1-associated MN. Notably, 20 of 26 evaluable patients had an immunological response with a decrease in anti-PLA2R1 autoantibody levels. In addition, the NewPLACE trial (NCT04733040) has completed recruitment and is looking at the efficacy and safety of felzartamab in patients with PLA2R1-assocaited MN, while the MONET trial (NCT04893096) is investigating felzartamab in patients with MN who are refractory to CD20-targeted treatment. The latter trial is active, but currently not recruiting. Together, these trials will have important implications in the treatment landscape of MN, as they will inform whether plasma cell targeting is a valid therapeutic strategy in both newly diagnosed patients as well as in patients with insufficient response to CD20-mediated B cell depletion.

**Table 1:** Completed, ongoing and terminated trials of plasma cell– (blue) and complement-targeted treatments (green) in MN.

Study	Molecule	Target	Study phase	Patient population	Endpoints	Current status
M-PLACE	Felzartamab	CD38	1b/2a	Newly diagnosed, relapsed and refractory MN	Incidence and severity of adverse events	Published
NewPLACE	Felzartamab	CD38	2	Anti-PLA2R-positive MN with indication for immunosuppressive treatment	Percent change of anti-PLA2R antibody levels	Completed
MONET	Felzartamab	CD38	2	MN patients who failed anti-CD20 treatment	Change in 24-hour urinary protein excretion	Active, not recruiting
N/A	Eculizumab	C5	N/A		N/A	Terminated due to lack of efficacy
N/A	Iptacopan	CFB	2	MN with anti-PLA2R >60 RU/ml	Ratio between baseline UPCR and UPCR at 24 weeks of treatment	Terminated by sponsor due to lack of efficacy
RENEW	BCX9930	CFD	2	New diagnosis of MN	Percent change from baseline in 24-hour UPCR at week 24	Terminated by sponsor
N/A	Narsoplimab	MASP2	2	New diagnosis of MN	Treatment-related adverse events	Unknown status

UPCR: urine protein:creatinine ratio.

The first trial looking at complement inhibition in MN investigated the efficacy of eculizumab, an anti-C5 monoclonal antibody (Fig. 3, right). This trial was halted early due a lack of efficacy, but the drug may have been underdosed in the context of severe proteinuria, with the loss of large amounts of plasma proteins into the urine, including the therapeutic antibody. More recently, iptacopan, a small molecule inhibitor of CFB, BCX 9930, an inhibitor of factor D, and narsoplimab, a monoclonal antibody against MASP2, were all investigated in clinical trials including patients with MN (NCT04154787, NCT05162066 and NCT02682407, respectively). However, the first two trials were terminated due to sponsor decision and the latter one has an unknown status, indicating that no results in regard to the efficacy of complement-targeted treatments will be revealed in the near future.

## CONCLUSIONS

From a pathophysiological standpoint, plasma cells as an important source of autoantibodies as well as the complement system as a key pathogenic effector mechanism undoubtedly qualify as therapeutic targets in patients with MN. However, studies investigating treatment efficacy in large numbers of patients are still lacking. The field is eagerly awaiting data on plasma cell depletion in patients with MN, either as a primary treatment or in patients refractory to CD20-targeted B cell depletion. Complement-targeted treatments, in contrast, seem to be failing. One could speculate that the damage to the kidney filtration barrier is not solely mediated by the complement system, a concept that is actually also supported by recent animal studies, where complement-targeted treatment attenuated but did not abrogate proteinuria [51, 52]. In the opinion of this author, the primary treatment goal in MN should be clearance of the autoantibodies, regardless of whether this is achieved through B cell or plasma cell depletion, as this represents the earliest interference in the pathogenic cascade. However, I can imagine a role for complement-targeted treatment, particularly in the context of uncontrolled and exacerbating nephrotic syndrome with complications such as thrombotic events. Currently there is no established way to determine which patients are most likely to respond to complement-targeted treatments in general or complement pathway-specific molecules in particular. One way of addressing this would be the evaluation of complement activity in the kidney tissue, enabling tailoring of the therapy to the dominant complement pathway (e.g. narsoplimab in case of lectin pathway activity and iptacopan in case of alternative pathway activity). In addition, newer complement inhibitors such as the anti-C5 antibody ravulizumab should be evaluated for their efficacy in patients with MN.

Regardless of the outcome, the systematic evaluation of plasma cell– and complement-targeted therapies definitely represents a step forward in regard to the future implementation of pathogenesis-based treatments in patients with MN. However, knowledge of the MN-specific autoantigens, with PLA2R1 leading the way, will enable the development of highly innovative and more specific treatments. This may involve the clearance of autoantibodies using antibody-like molecules that capture the autoantibodies in the circulation and target them to the liver for degradation [53, 54], the depletion of autoreactive B cells by chimeric autoantibody receptor T or natural killer cells [55] and the use of bispecific T cell engagers [56]. In the opinion of this author, these approaches represent new ways of pathogenesis-based disease targeting while sparing protective immunity with the potential to shape the treatment landscape of MN in the future.

## Data Availability

No new data were generated or analysed in support of this research.
